# Impact of Naturally Occurring Asbestos on Asbestos Ban: Regulations and Experience of the Republic of Korea

**DOI:** 10.3390/ijerph19020742

**Published:** 2022-01-10

**Authors:** Jiwoon Kwon

**Affiliations:** Occupational Safety and Health Research Institute, Korea Occupational Safety and Health Agency, 478-1 Munemi-ro, Bupyeong-gu, Incheon 21417, Korea; jwk@kosha.or.kr; Tel.: +82-32-510-0752

**Keywords:** naturally occurring asbestos, public policy, national experience, Korea

## Abstract

This review examined the main issues debated in Korea regarding the production and use of materials containing naturally occurring asbestos (NOA) as impurities, and investigated the impacts of these debates on the asbestos ban, as well as the future implications. In Korea, incidents associated with the production and use of NOA-contaminated talc powders, construction rocks, serpentinites, and dolomite rocks raised public concern and led to accelerating the ban on asbestos. The main controversies concern policies on appropriate asbestos content limits, whether materials containing a trace amount of NOA should be banned, and the control of materials with high human exposure risk. To address recurring controversies, the implementation of preventive measures to manage elongated mineral particles and the use of transmission electron microscopy for more sensitive analysis need to be discussed, along with reaching social agreement on the controversial policies. To minimize the potential exposure to asbestos that may occur during the production and use of industrial minerals in the future, it is necessary to apply occupational exposure control measures and monitor the health effects of the relevant population groups. These national policies on NOA should be prepared based on close collaboration and discussion with policymakers, industry stakeholders, and related academic experts.

## 1. Introduction

Asbestos is a term that refers to six types of hydrous magnesium silicate minerals that exhibit asbestiform, a fiber type with long and thin fibers and fiber bundles [1]. Commercially valuable asbestos has been used as a raw material for various products, such as insulations and friction materials, because it shows enough flexibility to be woven into threads with high flame retarding, chemical resistance, and thermal and electrical insulating properties.

From 1940 to 2008, before the ban on asbestos use, Korea’s cumulative import volume of asbestos was 1,796,152 tons [2]. Most of the asbestos was used from the 1970s onwards, when the economy began to grow rapidly, until the end of the 1990s, when the phased ban on asbestos began. In Korea, 1999 cases of malignant mesothelioma, of which more than 80% occurred due to asbestos exposure, were observed in Korea from 1999 to 2018, and it is predicted that the number will continue to increase for the next 20 years [2]. In Korea, the number of cases covered by Industrial Accident Compensation Insurance for occupational asbestos exposure has increased every year, reaching a peak of 108 cases in 2019, while the cumulative number from 2001 to 2019 was 507 [3].

Naturally occurring asbestos (NOA) is a term for asbestos contained in rocks and soils that occurs naturally in geological processes [4]. Huge reserves of commercially valuable asbestos are scarce, although smaller amounts are more commonly found in soils or rocks. The health risks of NOA are generated in the respiratory tract when airborne asbestos particles are inhaled. The dangers of asbestos exposure are well known in NOA areas such as mining, construction, and recreation sites, where human activities disturb asbestos. The risks of using products contaminated with NOA have not been clearly established, although the products can be unsafe if the user consistently disturbs the asbestos [5].

In South Korea, rocks that contain NOA are distributed over 5514 km^2^, which is 5.5% of the total land area of the country [6]. From the Japanese colonial era, 42 asbestos mines were developed nationwide and produced 417,842 tons of asbestos [7]. Asbestos mining in Korea was discontinued in the 1980s. However, as a result of a health impact survey conducted by the Ministry of Environment (MOE) since June 2009 on residents within 1 km radius of 14 abandoned asbestos mines located in Chungcheongnam-do, asbestosis was diagnosed in 179 of 4057 residents, and half of the asbestosis cases were known to have no work history related to asbestos [8]. Since then, the MOE has extensively investigated the asbestos contamination of soil and rocks in areas around abandoned asbestos mines, along with the health effects on residents. An activity-based risk assessment revealed non-negligible health risk to residents living in NOA-contaminated areas near abandoned asbestos mines in Korea [9].

The production and use of industrial minerals, along with a variety of asbestos-disrupting activities in NOA-contaminated areas, can result in occupational or environmental exposure to NOA. In Korea, the presence of NOA in imported rocks and rock powders was first known in 1998 but failed to attract public attention at that time [10]. The fact that materials containing NOA as impurities have been used for various industrial purposes since the 2000s has attracted public interest and concern in Korea, and asbestos-related incidents affected the policy regarding its ban. In 2009, Kim discussed an incident caused by baby powders contaminated with NOA in terms of the health risk of low levels of asbestos exposure and implemented financial compensation for asbestos victims [11]. Yoon et al. identified five different phases of asbestos bans in Korea through narrative analyses of historical national asbestos profiles, and stated that a series of NOA incidents influenced cultural changes, resulting in the establishment of policies on asbestos [12]. However, the details of major incidents and recurring controversies associated with the production and use of NOA-contaminated materials have not been discussed. In this study, the main incidents related to the production and use of NOA-containing materials in Korea were examined, and their influence on changes in the regulations implemented for asbestos bans and future tasks were investigated.

## 2. Regulations for Asbestos Bans

### 2.1. Occupational Safety and Health Act (OSH Act)

In Korea, the first regulation on the use of asbestos can be found in the OSH Act of the Ministry of Employment and Labor (MOEL; then the Ministry of Labor) (Table 1). The MOEL banned the use of crocidolite and amosite at worksites in May 1997 and clearly specified their material control limits as 1% by weight in June 1999 [13,14]. It banned the use of materials that contain more than 1% of anthophyllite, tremolite, and actinolite asbestos by weight in June 2003 [15]. Among the six types of asbestos, only chrysotile could be used at worksites under strict work environment management after approval from the government. In January 2007, regulations on asbestos-containing products were initiated to ban the production, import, and use of cement products and friction materials for automobiles with more than 1% of asbestos by weight [16]. In January 2008, the control limit for the asbestos content was decreased to 0.1% by weight, and the use of all asbestos-containing products was prohibited, although asbestos-containing gaskets, friction materials excluding those used for automobiles, and gaskets and insulation used for some chemical equipment and military supplies were exempted [17]. Asbestos-containing gaskets and friction materials excluding those used for automobiles were additionally banned from January 2009 [17]. In 2014, a survey was conducted on the use of asbestos-containing gaskets and insulation for certain chemical equipment and military supplies, which were exempted from the ban, and it was confirmed that all products were replaced with non-asbestos products [18]. Accordingly, the import, manufacture, and use of all products containing more than 1% of asbestos by weight were prohibited without exception from April 2015 [19]. The import and use of materials containing more than 1% of chrysotile by weight were outlawed in February 2016, as products were no longer manufactured by intentionally adding asbestos [20]. In Korea, the ban on all forms of asbestos and asbestos-containing products became effective in April 2016. However, asbestos-containing gaskets and insulation were used in limited quantities for certain chemical equipment and military supplies, which were set as exceptions to the ban after January 2009. Therefore, the ban on all forms of asbestos in Korea was practically initiated from January 2009.

### 2.2. Asbestos Safety Management Act

As interest in asbestos surged and exposure to asbestos from the soils and rocks contaminated with NOA became a social issue in Korea in the late 2000s, the MOE enacted the Asbestos Safety Management Act and implemented it in April 2012 to minimize environmental exposure to asbestos [21]. The MOE designated materials that are highly likely to contain asbestos with high risks for enforcement actions under the Asbestos Safety Management Act and set control limits for the asbestos content. When the act was first enacted in April 2012, talc, vermiculite, sepiolite, and serpentinite were designated as materials suspected of containing asbestos [22]. According to the Asbestos Safety Management Act, the import and production of materials suspected of containing more than 1% of asbestos are prohibited, and people who import or produce these materials must prove through sampling and analysis that these control limits are not exceeded and obtain the approval of the MOE. For imported or produced materials suspected of containing asbestos, the control limit is specified in detail considering the life cycle, physical form, and application [23]. The content control limit is 0.1% for products that use talc, vermiculite, sepiolite, or serpentinite as raw materials, and no asbestos must be detected for materials in powder form that are used in direct contact with the human body or as construction materials, such as those used in road surfaces or pavements. Furthermore, no asbestos must be detected on the surfaces of rocks that are used for construction, and the control limit for the remaining applications is 0.1%.

### 2.3. Pharmaceutical Affairs Act

The Ministry of Food and Drug Safety (MFDS) of Korea sets quality standards for raw materials to ensure the quality and safety of pharmaceutical and hygiene products in accordance with the Pharmaceutical Affairs Act. The incident caused by baby powders produced using asbestos-contaminated talc powders prompted the MFDS to add the test method and criteria of asbestos in talc to the Korean Pharmacopoeia [24]. The MFDS mandates that any asbestiform fiber should not be detected when talc powder samples are analyzed using polarized light microscopy (PLM) after analyzing asbestos using X-ray diffraction analysis (XRD) or infrared spectroscopy (IR).

## 3. Experience with NOA-Contaminated Materials in Korea

### 3.1. Asbestos-Contaminated Talc Powders

Talc is a phyllosilicate that has the chemical formula of Mg_3_Si_4_O_10_(OH)_2_ [26]. It is mainly processed into fine powders and used as a raw material for products in various industries, including pulp and paper manufacturing, plastics and rubber manufacturing, and in the pharmaceutical and cosmetics industries.

In some deposits of talc, tremolite or anthophyllite with the asbestiform crystal habit is found. The first incident caused by asbestos-contaminated talc powders in Korea was made public on 1 April 2009 by the media, who reported that asbestos was detected in five baby powder products out of the 12 available in the market. On the same day, the MFDS revealed that asbestos was detected in 11 baby powder products available in the market [27]. Sales of these products were prohibited from the next day, and those available in the market were recalled. It was discovered that products used talc powders that were contaminated with NOA as a raw material. On 3 April 2009, the MFDS added the “non-detection of asbestos” regulation to the quality standards of talc in the Korean Pharmacopoeia in accordance with the Pharmaceutical Affairs Act [24]. After finding that asbestos-contaminated talc was supplied to more than 300 companies as a raw material for cosmetics and pharmaceuticals, the MFDS banned the sale of five cosmetic products and 1122 pharmaceuticals and ordered a recall of the products available in the market [28].

The panic caused by asbestos-contaminated talc powders made Korean people aware of the hazard of asbestos, as it was detected in baby powders, cosmetics, and medicines commonly used by many people. Because of this incident, the understanding of the asbestos problem, which was mainly perceived as an occupational health problem occurring in workplaces where asbestos is handled as a raw material in the manufacture of products such as building materials, brake pads, and gaskets in Korea, was expanded to include environmental exposure. Follow-up media coverage reported that there are regulations prohibiting the use of asbestos contained in talc in Europe and Japan, which led to the criticism of the Korean government and expert groups for failing to recognize and respond to the possibility of talc-containing NOA in advance. When the MFDS conducted a survey on asbestos contained in baby powders, no asbestos was detected from imported baby powder products. The decision of the MFDS to prohibit sales of 1122 pharmaceutical products already available in the market was opposed by pharmaceutical companies due to a lack of evidence of risks. The MFDS carried out large-scale administrative measures to relieve public anxiety, although the health risk from taking pharmaceuticals that used asbestos-contaminated talc powders was low. Pharmaceutical companies had to accept losses due to the use of hazardous materials in their products, even though the raw materials were legally certified by the government, which led to lawsuits filed by pharmaceutical companies against the government. The incident was a starting point to discuss the management of NOA unintentionally contained in products in Korea. The comprehensive measures for asbestos management prepared by the National Policy Coordination Meeting of the government in July 2009 included content on the asbestos ban and NOA management as main topics, while the Asbestos Safety Management Act containing the discussed measures came into effect in April 2012 [21].

### 3.2. Asbestos-Contaminated Construction Rocks

On 10 February 2009, an NGO revealed that the construction rocks produced in a quarry near an abandoned asbestos mine in Chungcheongbuk-do were sold to more than 210 government-ordered construction sites across the country [29]. The geology of the area encompassing the quarry had Ordovician dolomite as the base, as well as tremolite and actinolite, which are calcium-containing amphiboles, present along the vein. Since the quarry was located approximately 1 km south of the main entrance to the abandoned asbestos mine along the southwestern slope of the mountain, it was estimated that the mine’s asbestos vein led to the quarry. Later, a survey by the MOE revealed that asbestiform tremolite was widely distributed in rocks and soils in this area. A debate emerged on the risks of construction rocks containing tremolite asbestos, which were sold throughout the country. Although the local government explained that the asbestos contained in the construction rocks had a low level of risk, those construction rocks installed in public facilities, such as trails and parks, were removed or an encapsulant was applied to prevent asbestos scattering.

The use of asbestos-contaminated construction rocks showed that it is necessary to examine the presence of NOA in areas where mineral resources are obtained. Since the quarry was in proximity to the abandoned asbestos mine and located on the opposite slope of the same mountain, the presence of asbestos was highly likely. However, this was not considered by the authorities when the quarry company obtained permission to mine for construction rocks. It was also discussed that the level of asbestos content control limits for rocks must be established and analyzed. Some believed that determining whether the asbestos content control limit had been exceeded was difficult, since analytical results varied significantly due to the non-uniform content of NOA even after specifying a set value. Additionally, the analysis of rocks through crushing could increase the detection limit because asbestos could be diluted with non-asbestos particles in the rock. The MOE specified that “no asbestos must be exposed on the surfaces of rocks.” An inspection method that used target sampling on parts where asbestos was suspected to be present on the rock surface was to be implemented as a standard for banning asbestos in construction rocks, as specified in the Asbestos Safety Management Act [23].

### 3.3. Asbestos-Contaminated Serpentinite

Serpentine is a group of magnesium silicate hydrate minerals with the chemical formula of Mg_3_Si_2_O_5_(OH)_4_, which exists in nature as lizardite, antigorite, or chrysotile [30]. Serpentinite refers to a rock mass that combines lizardite and antigorite in the form of fine powders. It is mainly used as an auxiliary material for steelmaking and as a raw material for fertilizer or building materials, such as stones used for decorative purposes.

Despite its various applications, serpentinite can contain chrysotile. The fact that chrysotile-contaminated serpentinite was used as an auxiliary material in three steel mills in Korea was revealed by an NGO on 27 January 2011 [31]. At that time, three mines in Korea were allowed by the Korean government to produce serpentinite rocks after being closed in the 1980s due to the ban on asbestos mining. After the revelation, one of the mines ceased mining activities and the serpentinite used in two steel mills was replaced with dolomite, although the remaining two mines and one steel mill continued the mining and use of serpentinite. In September of the same year, asbestos-contaminated serpentinite was revealed to be present in five baseball fields used by the Korean professional baseball league and eight school playgrounds [32,33]. Although the serpentinite used as a floor material was removed, concerns were raised over the health of workers who could be exposed to asbestos during the production, transport, and use of the serpentinite.

This incident caused controversy over the NOA control limit. Despite a demand that mines must be prevented from mining asbestos-contaminated serpentinite, there was no law applicable at that time. The OSH Act banned products that contain more than 0.1% of asbestos, although the MOEL interpreted this regulation as applicable only to processed products and not mined rocks [34]. The MOEL did not prohibit serpentinite mining and ordered that workers’ exposure to asbestos be monitored and health checks be performed on them. These measures were criticized as the government did not ban asbestos-containing minerals despite its ban on asbestos.

### 3.4. Asbestos-Contaminated Dolomite Rocks

Dolomite is an anhydrous carbonate mineral with the chemical formula of CaMg(CO_3_)_2_, and is a type of sedimentary rock [35]. Dolomite is used as a raw material for decorative stones, concrete aggregates, and fertilizers and as a source of magnesium in steelmaking.

Tremolite asbestos was detected in mortar available in the market on 21 September 2020, and the use of these products in newly built elementary schools and public buildings was revealed by an NGO [36]. It was found that these products were created from an NOA-containing dolomite obtained from a dolomite mine in Korea. After this revelation, the dolomite mine ceased mining activities and the production of dolomite-containing mortar was suspended, although it became necessary to manage the asbestos contained in the products already used in buildings. The relevant office of education conducted an asbestos survey on buildings where asbestos-contaminated mortar was used and decided to apply encapsulant to parts where asbestos can be scattered due to outer exposure.

This incident sparked a debate over minerals that must be included in materials suspected of containing asbestos, which are managed under the Asbestos Safety Management Act of Korea. Although this act prohibited the use of materials suspected of containing more than 1% of asbestos and set the control limit for products to non-detection of asbestos or 0.1%, dolomite was not a material suspected of containing asbestos according to the act. Therefore, 1% was applied as the control limit for asbestos-containing products according to the OSH Act at that time. Although dolomite was one of the 12 candidate minerals suspected of containing asbestos in the expert forum held by the MOE to enact the Asbestos Safety Management Act, industries strongly opposed this because dolomite was a mineral produced in large quantities in Korea. Most of the controversial mortar products were not banned as asbestos-containing products in the OSH Act because less than 1% of tremolite was detected, so it was asserted that the asbestos control limit should be reduced to 0.1%. The classification of rocks was also controversial. The rocks used in mortar were dolomitic limestone when classified mineralogically, and there was an opinion from the mineralogical society that they cannot be simply classified into either dolomite or limestone.

## 4. Discussion

In light of the purpose of the OSH Act to manage the occupational exposure of workers to hazardous materials, the ban on asbestos under the OSH Act basically considered the use of mine-grade asbestos as raw material for products at worksites. The ban on NOA unintentionally contained as impurities in products under the OSH Act was examined only after the emergence of problems with the use of talc and tremolite rocks contaminated with asbestos in 2009. The MOEL mentioned in a Q&A session in 2010 that rocks were not banned because they were not products and that processed rocks were the target of the ban under the OSH Act [34]. This made it clear that mining of NOA-containing rocks was not prohibited but the production and use of products containing NOA in excess of the control limit was banned under the OSH Act. As for cases in other countries, the Ministry of Health, Labour, and Welfare of Japan banned materials that contain more than 0.1% of asbestos by weight regardless of unintentionally contained asbestos impurities under the OSH Act in 2006 [37]. While Korea and Japan set certain control limits for the asbestos content and ban materials that contain NOA in excess of such limits, the United Kingdom and European Union prohibit intentionally contained asbestos but permit NOA as exceptions [38,39]. For these exceptions, the European Union mentioned that a ban on NOA may prohibit the mining and use of various mineral resources because a small amount of asbestos is contained as impurities in the bedrocks of countries [40]. Considering the hazards of asbestos, it would be ideal to ban all materials where asbestos is detected. However, considering the value of minerals used as resources in various industries and products, it is practically impossible to ban all rocks containing asbestos as impurities regardless of the asbestos content. To minimize unnecessary social controversy due to NOA after the implementation of the asbestos ban, a national policy on the ban on asbestos needs to be prepared through careful discussion among stakeholders on the mining and use of rocks that may contain NOA.

Setting a cut-off for prohibiting hazardous materials is the same as determining the maximum content of such materials that can be legally used. The best method to set these control limits is to determine them by evaluating the level of harmlessness to health, although this level for asbestos is still not known. In Korea, the control limit for asbestos-containing materials that allows the import and production of such materials is 1% under the OSH and the Asbestos Safety and Management Acts [19,22]. Cases of the intentional addition of less than 1% of asbestos were rarely found in adhesives, sealing materials, and fillers, and cases of the intentional addition of less than 0.1% of asbestos in the manufacture of products were not reported [41]. Ki et al. conducted a survey on asbestos content in products of certain companies in Korea and found no case where less than 1% of asbestos was present [42]. Therefore, the 1% criterion bans most materials in which asbestos is intentionally added and those in which more than 1% of asbestos is unintentionally added as impurities, and allows the use of materials with very small amounts (less than 1%) of asbestos. In Korea, there was criticism that the 1% criterion set by the MOE as the asbestos content for banning the import and production of materials suspected of containing asbestos in 2012 under the Asbestos Safety Management Act cannot block the import and production of NOA-contaminated materials. In the NOA-contaminated serpentinite case in 2011, the average asbestos content in the crushed serpentinite produced from two quarries was 0.01 and 0.11%, respectively, which indicated that one was lower than 0.1% and the other was between 0.1 and 1% [43]. Regarding the use of asbestos-contaminated dolomite in 2020, less than 1% of asbestos was detected in most mortar samples. Therefore, an opinion was raised that the asbestos content control limit must be lowered to 0.1% for all materials because the use of minerals containing small amounts of NOA as raw materials for products further decreased the asbestos content in the products through dilution, although it was not accepted by the MOEL. The MOEL changed its standard for asbestos content from 0.1% to 1% in April 2015 because it intended to seek legal harmony with the OSH Act, the upper provision that bans substances containing more than 1% asbestos, and it intended to exclude rocks containing less than 1% NOA from the ban. Among materials that may contain NOA, the MOE set the control limit to 0.1% or non-detection of asbestos for products containing four minerals, which need to be managed due to the high usage and high possibility of human exposure to asbestos; this was done instead of applying the 0.1% criterion to all materials [23]. The quality standard of non-detection of asbestos set by the MFDS for talc applies only to the raw materials used in the target products of the Pharmaceutical Affairs Act, such as pharmaceutical and hygiene products [24]. Setting strict criteria considering the usage, method of use, and applications of minerals that may contain NOA, and prohibiting minerals with high risks are a few of the methods that can be considered when preparing an asbestos ban.

Amphiboles, which commonly exist in rocks as naturally occurring impurities, are present in various crystal habits, which include the prismatic and asbestiform depending on the mineral generation conditions [44]. However, since there is no scientific consensus for distinguishing asbestiform from other fibrous forms, there have been debates over the analytical results regarding asbestos and the toxicity of non-asbestiform minerals [5,45,46,47]. In particular, when amphiboles unintentionally contained in rocks are analyzed, the distinction between asbestiform and non-asbestiform minerals is still obscure from time to time. In Korea, the MOEL and MFDS referred to typical characteristics of asbestiform minerals seen in the optical microscope determined by the recommended analytical method of the International Organization for Standardization or United States Environmental Protection Agency [41,48]. However, the Asbestos Safety Management Act from the MOE specified an aspect ratio of 3:1 or more as the morphological characteristic for distinguishing asbestos, in the same manner as the rule for counting airborne fibers with phase contrast microscopy [25]. Therefore, unlike the MOEL and MFDS, for the MOE, the definition of asbestos covers six types of mineral fibers that encompass both asbestiform and non-asbestiform fibers. To minimize the controversy that may occur in the future due to NOA-containing minerals, it is necessary to unify asbestiform analytical standards so that uniform analytical results can be derived for the same sample in the future. In France, after an extensive review on the cleavage fragment issue, the government concluded that national agreement on the terminology of elongate mineral particles (EMPs) was required to address recurring NOA controversies [47]. EMPs was the term first used by the National Institute for Occupational Safety and Health to refer to thoracic-sized mineral fibers occurring in either an asbestiform habit or a non-asbestiform habit [49]. Particularly, a public discussion is required on whether to manage EMPs in a precautionary manner together with asbestos, considering the unclear asbestiform analytical criteria at present and the potential toxicity of EMPs.

Despite the ban on asbestos-containing materials, the production and use of materials containing NOA at concentrations below the regulatory control limit or detection limit of the analytical method might result in significant exposure to airborne asbestos fibers. The potential asbestos exposure problem caused by the limited asbestos analysis technology is well known in the case of talc powders. For the analysis of asbestos in bulk samples, PLM, XRD, IR, or transmission electron microscopy (TEM) is widely used. In PLM, fine asbestos particles are not visible due to the low resolution of the light-optical system [50]. XRD can detect up to 0.1% of tremolite, 0.25% of chrysotile, and 2% of anthophyllite by weight in talc powders [50], while IR can detect up to 0.1% of asbestos by weight [51]. On the other hand, TEM is a sensitive analytical technology used for detecting minute amounts of asbestos in talc due to its high resolution and low limit of detection. Recently, when talc-based cosmetics available in the U.S. market were analyzed using TEM, asbestos was detected at low levels in products for which no asbestos was detected through PLM [52]. The number of chrysotile fibers present at the lowest level of detection by XRD can be about 10^9^ fibers per milligram of talc powders when analyzed by TEM, which might lead to significant exposure to users of talc end products [50]. Several studies reported that workers who mine or process minerals containing NOA as impurities or those who use them as raw materials can be exposed to concentrations that exceed the occupational exposure limit of asbestos [53,54,55]. In 2011, the serpentinite produced from NOA-contaminated serpentine mines in Korea exhibited an asbestos content far less than the 1% criterion, although the maximum concentration of asbestos in the air to which workers were exposed was 0.076 f/cc, which was 76% of the exposure limit [43]. Therefore, regardless of the asbestos ban, national policies to manage asbestos exposure that may occur from materials contaminated with NOA should be implemented to minimize the occurrence of asbestos-related diseases.

## 5. Conclusions

Korea’s experiences regarding NOA demonstrate that when implementing asbestos bans, the production, import, and use of rocks unintentionally containing NOA as well as the intentional use of asbestos must be considered in order to reduce unnecessary controversies and public concern regarding asbestos. To address recurring controversies over the production and use of NOA-containing materials, policies on whether materials containing a trace amount of NOA should be banned, appropriate asbestos content permissible limits, and the control of materials with high human exposure risk should be established in consideration of national circumstances. It is necessary to discuss whether the asbestos ban will be extended to EMPs from a preventive point of view and the use of TEM for more sensitive analysis of NOA. To minimize future asbestos-related diseases, policies to manage exposure to NOA, such as the application of engineering controls, use of personal protection equipment, periodic asbestos concentration monitoring, health checks, and warnings against the possibility that a material contains asbestos, are required along with the asbestos ban. Continuous surveillance and ad hoc studies in populations particularly exposed to the problem of NOA should be implemented to monitor possible adverse health effects of NOA. These national policies on NOA should be established based on close cooperation and examinations from industry stakeholders and policymakers, together with experts in the fields of industrial hygiene, environmental science, health science, and mineralogy.

## Figures and Tables

**Table 1:** Regulations for asbestos bans in Korea.

Date	Regulations	Analytical Methods	Authorities	References
16 May 1997	The import, transfer, and use of amosite or crocidolite are prohibited	Not specified	MOL ^4^	[13]
8 June 1999	The production, import, transfer, and use of materials containing more than 1% of amosite or crocidolite by weight are prohibited.	Not specified	MOL	[14]
30 June 2003	The production, import, transfer, and use of materials containing more than 1% of anthophyllite, tremolite, or actinolite asbestos by weight are prohibited.	Not specified	MOL	[15]
1 January 2007	The production, import, transfer, and use of cement products and friction materials for automobiles containing more than 1% of asbestos by weight are prohibited.	Not specified	MOL	[16]
1 January 2008	The production, import, transfer, and use of all products containing more than 0.1% of asbestos by weight are prohibited. - The ban is suspended for gaskets, friction materials other than those for automobiles, and gaskets and insulation used in some chemical equipment and military supplies.	Not specified	MOL	[17]
1 January 2009	The production, import, transfer, and use of gaskets and friction materials other than those for automobiles that contain more than 0.1% of asbestos by weight are additionally prohibited.	Not specified	MOL	[17]
3 April 2009	The application of talc in which asbestos is detected in pharmaceutical and hygiene products is prohibited.	XRD ^1^ or IR ^2^ following PLM ^3^	MFDS ^5^	[21]
29 April 2012	The import and sales of materials suspected of containing asbestos (talc, vermiculite, sepiolite, and serpentinite) in excess of the following content criteria are prohibited. - For import or production: 1% - For use as a raw material for products: 0.1% - For use as powders, in road surfaces or pavements, or in contact with the human body—non-detection of asbestos - For rocks used in construction sites—non-detection of asbestos on the surface - For other applications: 0.1%	PLM for construction rocks, XRD following PLM for other applications	MOE ^6^	[22,23,24,25]
1 April 2015	The production, import, transfer, and use of all products containing more than 1% of asbestos by weight are prohibited.	Not specified	MOEL ^7^	[19]
17 February 2016	The production, import, transfer, and use of materials containing more than 1% of chrysotile by weight are prohibited.	Not specified	MOEL	[20]

^1^ X-ray diffraction analysis. ^2^ Infrared spectroscopy. ^3^ Polarized light microscopy. ^4^ Ministry of Labor. ^5^ Ministry of Food and Drug Safety. ^6^ Ministry of Environment. ^7^ Ministry of Employment and Labor.
